# (2-Amino­ethane­thiol­ato-κ^2^ *N*,*S*)bis­[1,2-bis­(diphenyl­phosphan­yl)ethane-κ^2^ *P*,*P*′]ruthenium(II) hexa­fluoridophosphate

**DOI:** 10.1107/S1600536812044273

**Published:** 2012-10-31

**Authors:** Asako Igashira-Kamiyama, Motoshi Tamura, Takumi Konno

**Affiliations:** a Department of Chemistry, Graduate School of Science, Osaka University, Toyonaka, Osaka 560-0043, Japan

## Abstract

In the crystal of the title compound, [Ru(C_2_H_6_NS)(C_26_H_24_P_2_)_2_]PF_6_, the Ru^II^ atom is in a slightly distorted octa­hedral geometry, coordinated by one 2-amino­ethane­thiol­ate (aet) and two 1,2-bis­(diphenyl­phosphan­yl)ethane (dppe) ligands. The crystal consists of a pair of enanti­omers (Δ and Λ) of the compound. The Δ and Λ isomers have the λ and δ conformations for the aet chelate rings and the δ and λ conformations for the dppe chelate rings. The F atoms of the PF_6_^−^ counter-anion are disordered over three positions, with site occupancies of 0.4, 0.3 and 0.3.

## Related literature

For closely related structures, see: Tamura *et al.* (2007 ▶); Matsuura *et al.* (2006 ▶); Hanif *et al.* (1999 ▶). For conformation descriptors of the chelate rings, see: Gispert (2008 ▶). For the starting material, see: Bautista *et al.* (1991 ▶). For a description of the Cambridge Structural Database, see: Allen (2002 ▶).
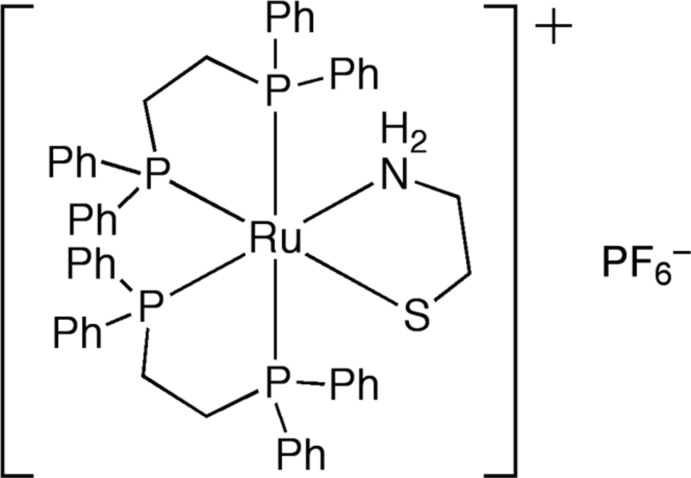


## Experimental

### 

#### Crystal data

[Ru(C_2_H_6_NS)(C_26_H_24_P_2_)_2_]PF_6_
*M* *_r_* = 1118.96
Monoclinic, 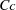

*a* = 21.1985 (17) Å
*b* = 11.4000 (9) Å
*c* = 20.9346 (17) Å
β = 106.588 (2)°
*V* = 4848.6 (7) Å^3^
*Z* = 4
Mo *K*α radiation
μ = 0.59 mm^−1^
*T* = 200 K
0.15 × 0.08 × 0.08 mm

#### Data collection

Rigaku R-AXIS RAPID diffractometer
Absorption correction: multi-scan (*ABSCOR*; Rigaku, 1995 ▶) *T*_min_ = 0.684, *T*_max_ = 0.954
23148 measured reflections
9513 independent reflections
7722 reflections with *I* > 2σ(*I*)
*R*_int_ = 0.067

#### Refinement

*R*[*F* ^2^ > 2σ(*F* ^2^)] = 0.044
*wR*(*F* ^2^) = 0.112
*S* = 1.20
9513 reflections
638 parameters
4 restraints
H atoms treated by a mixture of independent and constrained refinement
Δρ_max_ = 1.02 e Å^−3^
Δρ_min_ = −0.88 e Å^−3^
Absolute structure: Flack (1983 ▶), 3981 Friedel pairs
Flack parameter: −0.02 (3)

### 

Data collection: *PROCESS-AUTO* (Rigaku, 2000 ▶); cell refinement: *PROCESS-AUTO*; data reduction: *PROCESS-AUTO*; program(s) used to solve structure: *SHELXS97* (Sheldrick, 2008 ▶); program(s) used to refine structure: *SHELXL97* (Sheldrick, 2008 ▶); molecular graphics: *Yadokari-XG 2009* (Kabuto *et al.*, 2009 ▶); software used to prepare material for publication: *Yadokari-XG 2009*.

## Supplementary Material

Crystal structure: contains datablock(s) I, global. DOI: 10.1107/S1600536812044273/gk2528sup1.cif

Structure factors: contains datablock(s) I. DOI: 10.1107/S1600536812044273/gk2528Isup2.hkl

Additional supplementary materials:  crystallographic information; 3D view; checkCIF report

## Figures and Tables

**Table 1:** Selected bond lengths (Å)

Ru1—N1	2.209 (5)
Ru1—P1	2.3586 (14)
Ru1—P4	2.3672 (15)
Ru1—P3	2.3698 (13)
Ru1—P2	2.4249 (13)
Ru1—S1	2.4317 (15)

**Fig. 1.**
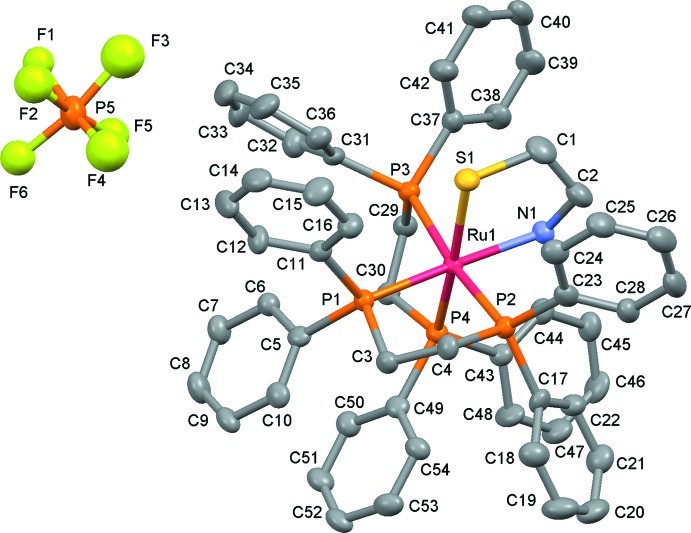
A view of the molecular structure of [Ru(aet)(dppe)2]PF6 with the atom-numbering scheme. H atoms and F atoms of the PF6- with minor occupancies were omitted for clarity. Ellipsoids represent 50% probability.

**Fig. 2.**
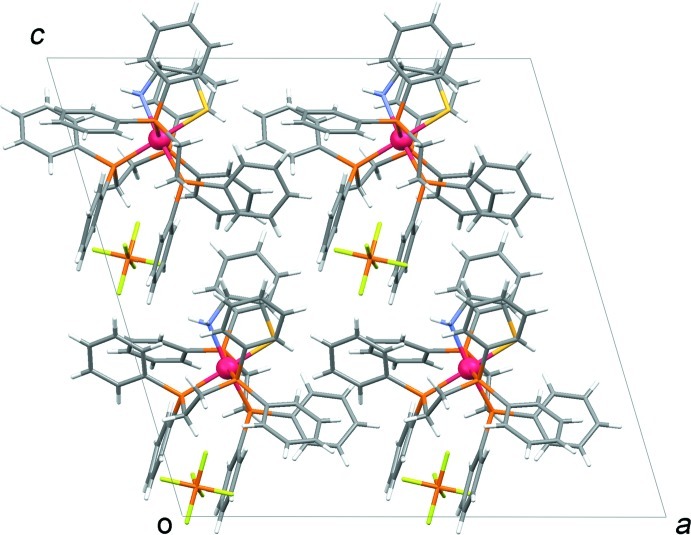
Crystal packing of [Ru(aet)(dppe)2]PF6 viewed along the y axis. F atoms of the PF6- with minor occupancies were omitted for clarity.

## supplementary crystallographic information

### Comment

It has been recognized that the preparation of ruthenium complexes having non-bridging aliphatic thiolato group(*s*) is difficult because of the high reactivity of a thiolato group bound to a metal center. For example, the direct reaction of Ru^II^ with 2-aminoethanethiol (Haet) led to the formation of thiolato-bridged trinuclear complex, [Ru{Ru(aet)_3_}_2_]^2+^ (Matsuura *et al.*, 2006), and furthermore, the reaction of [Ag{Ru(aet)(bpy)_2_}_2_]^3+^ (bpy = 2,2'-bipyridine) with HCl did not give an expected mononuclear complex, [Ru(aet)(bpy)_2_]^+^, but produced a dinuclear complex with a disulfide bond, [Ru_2_(cysta)(bpy)_4_]^4+^ (cysta = cystamine) (Tamura *et al.*, 2007). Here, we report the synthesis and crystal structure of [Ru(aet)(dppe)_2_]PF_6_, which is a quite rare example of a crystallographically characterized ruthenium(II) complex with an aliphatic thiolato donor. The use of the bulky diphenylphosphine ligand seems to be responsible for the successful isolation of this compound.

The reaction of [RuCl_2_(dppe)_2_] (Bautista *et al.*, 1991) with excess Haet in methanol in the presence of NH_4_PF_6_ gave a yellow powder of [Ru(aet)(dppe)_2_]PF_6_. Single-crystals suitable for X-ray analysis were obtained by the recrystallization of the yellow powder from methanol.

The compound crystallized in a non-centrosymmetric space group *Cc*, the asymmetric unit of which contains one complex cation and one PF_6_^-^ anion. The Ru atom is in an NP_4_S octahedral geometry coordinated by two dppe-*κP*,*P* and one aet-*κN*,*S* ligands (Fig. 1). The Ru–S [2.431 (2) Å] and Ru–N [2.212 (5) Å] bond distances are slightly longer than those of the related ruthenium(II) complexes with aet ligand(*s*) (Ru–S = 2.291–2.394 Å, Ru–N = 2.133–2.212 Å) (Tamura *et al.*, 2007; Matsuura *et al.*, 2006; Hanif *et al.*, 1999). On the other hand, the Ru–P bond distances (average 2.380 Å) are similar to those of ruthenium(II) complexes with dppe ligands (av. 2.32 Å), as found in the Cambridge Structural Database (Allen, 2002). Consistent with the space group *Cc*, the crystal consists of a pair of enantiomers (Δ and Λ) of the ruthenium(II) complex. Two dppe P,P-chelate rings in the complex adopt an *ob* conformation (δ for Δ isomer, λ for Λ isomer), while its N,S-aet chelate ring has a *lel* conformation (λ for Δ isomer, δ for Λ isomer). It may be interesting to note that no significant specific intermolecular interactions have been found, except for very weak N-H···F interactions between the complex cations and PF_6_^-^ anions (Fig. 2).

### Experimental

To a solution of 0.20 g (0.21 mmol) of [RuCl_2_(dppe)_2_] in 200 ml of methanol was added 0.078 g (1.01 mmol) of Haet and 0.338 g (2.07 mmol) of NH_4_PF_6_. The mixture was stirred at room temperature for 1 h. The resulting yellow suspension was concentrated to dryness with a rotary evaporator. The residue was washed with water to give a yellow powder of [Ru(aet)(dppe)_2_]PF_6_.1.5H_2_O. Yield 0.178 g (76%). Anal. Calcd for [Ru(aet)(dppe)_2_]PF_6_.1.5H_2_O: C, 58.54; H, 4.64; N, 1.26%. Found: C, 58.73; H, 4.91; N, 1.16%. ^1^H NMR (400 MHz, DMSO-*d*_6_): *d*, 8.04 (2*H*, t, *J* = 8.4 Hz), 7.93 (2*H*, t, *J* = 7.9 Hz), 7.72–7.64 (5*H*, m), 7.54 (2*H*, t, *J* = 8.5 Hz), 7.44–7.25 (17*H*, m), 7.05 (2*H*, t, *J* = 7.0 Hz), 6.98–6.89 (6*H*, m), 6.57 (2*H*, t, *J* = 8.3 Hz), 6.31 (2*H*, t, *J* = 8.4 Hz), 2.92–2.82 (3*H*, m), 2.38–2.34 (2*H*, m), 2.10–2.07 (2*H*, br m), 1.58 (1*H*, br s), 1.39 (1*H*, br s), 1.06 (1*H*, br s), 0.86 (1*H*, br s), 0.67 (1*H*, br s). ^31^P NMR (202.47 MHz, DMSO-*d*_6_): *d*, p.p.m. 50.25 (1P, d, *J* = 321.8 Hz), 48.79 (1P, s), 47.34 (1P, s), 29.74 (1P, d, *J* = 317.1 Hz). IR (KBr, cm^-1^): 1435 (*ν*_Ph_), 1097 and 745–694 (*ν*_P—Ph_), 839 (PF_6_^-^), 557 (PF_6_^-^).

Single crystals of [Ru(aet)(dppe)_2_]PF_6_ suitable for X-ray analysis were obtained by the recrystallization of a yellow powder from methanol at room temperature.

### Refinement

H atoms bound to C atoms were placed at calculated positions [C—H = 0.99 (methylene) and 0.95 (phenyl)] and refined as riding with isotropic displacement parameters [*U*_iso_(H) = 1.2*U*_eq_(C)]. H atoms bound to N atoms were located in a difference Fourier map and refined with distance restraint and constrained displacement parameters [N—H = 0.89 (2) Å, *U*_iso_(H) = 1.5*U*_eq_(N)]. The F atoms of the hexafluoridophosphate are disordered over three positions (F1–F6, F7–F12 and F13–F18) with site occupancies of 0.4 (F1–F6) and 0.3 (F7–F12, F13-F18), and refined isotropically.

### Crystal data

**Table:**

[Ru(C_2_H_6_NS)(C_26_H_24_P_2_)_2_]PF_6_	*F*(000) = 2296
*M**_r_* = 1118.96	*D*_x_ = 1.533 Mg m^−^^3^
Monoclinic, *C**c*	Mo *K*α radiation, λ = 0.71075 Å
Hall symbol: C -2yc	Cell parameters from 15716 reflections
*a* = 21.1985 (17) Å	θ = 3.1–27.4°
*b* = 11.4000 (9) Å	µ = 0.59 mm^−^^1^
*c* = 20.9346 (17) Å	*T* = 200 K
β = 106.588 (2)°	Prism, yellow
*V* = 4848.6 (7) Å^3^	0.15 × 0.08 × 0.08 mm
*Z* = 4	

### Data collection

**Table:**

Rigaku R-AXIS RAPID diffractometer	9513 independent reflections
Radiation source: fine-focus sealed tube	7722 reflections with *I* > 2σ(*I*)
Graphite monochromator	*R*_int_ = 0.067
Detector resolution: 10.00 pixels mm^-1^	θ_max_ = 27.5°, θ_min_ = 3.1°
ω scans	*h* = −27→27
Absorption correction: multi-scan (*ABSCOR*; Rigaku, 1995)	*k* = −14→14
*T*_min_ = 0.684, *T*_max_ = 0.954	*l* = −27→26
23148 measured reflections	

### Refinement

**Table:**

Refinement on *F*^2^	Secondary atom site location: difference Fourier map
Least-squares matrix: full	Hydrogen site location: inferred from neighbouring sites
*R*[*F*^2^ > 2σ(*F*^2^)] = 0.044	H atoms treated by a mixture of independent and constrained refinement
*wR*(*F*^2^) = 0.112	*w* = 1/[σ^2^(*F*_o_^2^) + (0.0354*P*)^2^ + 0.1*P*] where *P* = (*F*_o_^2^ + 2*F*_c_^2^)/3
*S* = 1.20	(Δ/σ)_max_ < 0.001
9513 reflections	Δρ_max_ = 1.02 e Å^−^^3^
638 parameters	Δρ_min_ = −0.88 e Å^−^^3^
4 restraints	Absolute structure: Flack (1983), 3981 Friedel pairs
Primary atom site location: structure-invariant direct methods	Flack parameter: −0.02 (3)

### Special details

**Table:**

Geometry. All e.s.d.'s (except the e.s.d. in the dihedral angle between two l.s. planes) are estimated using the full covariance matrix. The cell e.s.d.'s are taken into account individually in the estimation of e.s.d.'s in distances, angles and torsion angles; correlations between e.s.d.'s in cell parameters are only used when they are defined by crystal symmetry. An approximate (isotropic) treatment of cell e.s.d.'s is used for estimating e.s.d.'s involving l.s. planes.
Refinement. Refinement of *F*^2^ against ALL reflections. The weighted *R*-factor w*R* and goodness of fit *S* are based on *F*^2^, conventional *R*-factors *R* are based on *F*, with *F* set to zero for negative *F*^2^. The threshold expression of *F*^2^ > σ(*F*^2^) is used only for calculating *R*-factors(gt) *etc*. and is not relevant to the choice of reflections for refinement. *R*-factors based on *F*^2^ are statistically about twice as large as those based on *F*, and *R*-factors based on ALL data will be even larger.

### Fractional atomic coordinates and isotropic or equivalent isotropic displacement parameters (Å^2^)

**Table:**

	*x*	*y*	*z*	*U*_iso_*/*U*_eq_	Occ. (<1)
Ru1	0.183683 (18)	0.68842 (3)	0.323758 (19)	0.02092 (10)	
S1	0.29912 (7)	0.67929 (12)	0.38742 (8)	0.0301 (4)	
N1	0.1722 (2)	0.6281 (4)	0.4201 (2)	0.0302 (10)	
H1	0.137 (2)	0.654 (5)	0.432 (3)	0.045*	
H2	0.167 (3)	0.550 (2)	0.425 (3)	0.045*	
C1	0.2930 (3)	0.6267 (6)	0.4680 (3)	0.0429 (16)	
H3	0.3015	0.5412	0.4710	0.052*	
H4	0.3278	0.6649	0.5039	0.052*	
C2	0.2286 (3)	0.6491 (5)	0.4800 (3)	0.0391 (14)	
H5	0.2272	0.7315	0.4945	0.047*	
H6	0.2241	0.5977	0.5165	0.047*	
P1	0.20553 (6)	0.77721 (11)	0.23054 (7)	0.0232 (3)	
C3	0.1951 (3)	0.9383 (4)	0.2376 (3)	0.0271 (12)	
H7	0.2191	0.9798	0.2101	0.032*	
H8	0.1479	0.9587	0.2203	0.032*	
C4	0.2210 (3)	0.9779 (4)	0.3095 (3)	0.0280 (12)	
H9	0.2097	1.0614	0.3131	0.034*	
H10	0.2695	0.9704	0.3243	0.034*	
P2	0.18515 (6)	0.88868 (11)	0.36321 (7)	0.0242 (3)	
C5	0.1565 (2)	0.7499 (4)	0.1439 (3)	0.0259 (12)	
C6	0.1508 (2)	0.6354 (4)	0.1183 (3)	0.0263 (12)	
H11	0.1718	0.5727	0.1463	0.032*	
C7	0.1152 (3)	0.6119 (5)	0.0530 (3)	0.0347 (13)	
H12	0.1113	0.5336	0.0367	0.042*	
C8	0.0855 (3)	0.7025 (5)	0.0118 (3)	0.0401 (14)	
H13	0.0611	0.6867	−0.0331	0.048*	
C9	0.0910 (3)	0.8159 (5)	0.0354 (3)	0.0324 (13)	
H14	0.0708	0.8782	0.0066	0.039*	
C10	0.1258 (3)	0.8400 (5)	0.1010 (3)	0.0293 (12)	
H15	0.1289	0.9185	0.1169	0.035*	
C11	0.2893 (2)	0.7749 (4)	0.2191 (3)	0.0242 (11)	
C12	0.3020 (3)	0.7339 (5)	0.1614 (3)	0.0343 (14)	
H16	0.2668	0.7031	0.1265	0.041*	
C13	0.3647 (3)	0.7372 (6)	0.1539 (3)	0.0407 (16)	
H17	0.3723	0.7072	0.1144	0.049*	
C14	0.4161 (3)	0.7835 (5)	0.2031 (3)	0.0381 (15)	
H18	0.4591	0.7860	0.1978	0.046*	
C15	0.4046 (3)	0.8260 (5)	0.2602 (4)	0.0407 (15)	
H19	0.4396	0.8602	0.2939	0.049*	
C16	0.3418 (3)	0.8194 (5)	0.2691 (3)	0.0345 (13)	
H20	0.3350	0.8456	0.3097	0.041*	
C17	0.1085 (3)	0.9649 (4)	0.3618 (3)	0.0300 (12)	
C18	0.0946 (3)	1.0811 (5)	0.3404 (3)	0.0414 (15)	
H21	0.1262	1.1246	0.3260	0.050*	
C19	0.0363 (3)	1.1332 (5)	0.3400 (4)	0.0476 (17)	
H22	0.0280	1.2121	0.3254	0.057*	
C20	−0.0103 (3)	1.0720 (6)	0.3606 (4)	0.0460 (17)	
H23	−0.0510	1.1081	0.3594	0.055*	
C21	0.0020 (3)	0.9582 (5)	0.3828 (3)	0.0417 (15)	
H24	−0.0295	0.9164	0.3982	0.050*	
C22	0.0606 (3)	0.9053 (5)	0.3826 (3)	0.0314 (13)	
H25	0.0683	0.8262	0.3969	0.038*	
C23	0.2367 (3)	0.9304 (4)	0.4463 (3)	0.0304 (12)	
C24	0.3053 (3)	0.9331 (4)	0.4623 (3)	0.0350 (14)	
H26	0.3257	0.9182	0.4282	0.042*	
C25	0.3442 (3)	0.9567 (5)	0.5257 (3)	0.0416 (15)	
H27	0.3907	0.9586	0.5346	0.050*	
C26	0.3164 (3)	0.9775 (5)	0.5764 (3)	0.0435 (16)	
H28	0.3433	0.9914	0.6205	0.052*	
C27	0.2482 (3)	0.9779 (5)	0.5621 (3)	0.0455 (16)	
H29	0.2284	0.9956	0.5964	0.055*	
C28	0.2089 (3)	0.9527 (4)	0.4985 (3)	0.0336 (13)	
H30	0.1624	0.9505	0.4901	0.040*	
P3	0.19576 (6)	0.48846 (10)	0.29860 (7)	0.0233 (3)	
C29	0.1124 (3)	0.4253 (4)	0.2654 (3)	0.0277 (13)	
H31	0.1147	0.3537	0.2393	0.033*	
H32	0.0950	0.4028	0.3028	0.033*	
C30	0.0667 (2)	0.5138 (4)	0.2212 (3)	0.0272 (12)	
H33	0.0208	0.4846	0.2103	0.033*	
H34	0.0785	0.5229	0.1790	0.033*	
P4	0.07221 (7)	0.65771 (13)	0.26321 (8)	0.0236 (3)	
C31	0.2428 (3)	0.4391 (4)	0.2423 (3)	0.0276 (12)	
C32	0.2228 (3)	0.3441 (5)	0.2001 (3)	0.0420 (15)	
H35	0.1823	0.3059	0.1973	0.050*	
C33	0.2627 (3)	0.3046 (6)	0.1613 (4)	0.0479 (17)	
H36	0.2484	0.2415	0.1310	0.057*	
C34	0.3218 (3)	0.3564 (6)	0.1672 (3)	0.0496 (17)	
H37	0.3495	0.3269	0.1423	0.060*	
C35	0.3420 (3)	0.4508 (5)	0.2086 (4)	0.0449 (17)	
H38	0.3830	0.4872	0.2113	0.054*	
C36	0.3034 (3)	0.4934 (5)	0.2464 (3)	0.0337 (13)	
H39	0.3176	0.5589	0.2750	0.040*	
C37	0.2340 (3)	0.3912 (4)	0.3691 (3)	0.0301 (13)	
C38	0.1971 (3)	0.3350 (4)	0.4064 (3)	0.0375 (14)	
H40	0.1508	0.3452	0.3950	0.045*	
C39	0.2292 (3)	0.2641 (5)	0.4604 (4)	0.0464 (17)	
H41	0.2041	0.2253	0.4851	0.056*	
C40	0.2959 (4)	0.2496 (5)	0.4783 (3)	0.0465 (17)	
H42	0.3168	0.2019	0.5155	0.056*	
C41	0.3328 (3)	0.3040 (5)	0.4426 (4)	0.0454 (16)	
H43	0.3791	0.2930	0.4544	0.054*	
C42	0.3018 (3)	0.3752 (5)	0.3890 (3)	0.0393 (15)	
H44	0.3277	0.4140	0.3652	0.047*	
C43	0.0088 (2)	0.6345 (4)	0.3075 (3)	0.0265 (12)	
C44	0.0192 (3)	0.5576 (5)	0.3614 (3)	0.0318 (13)	
H45	0.0601	0.5176	0.3761	0.038*	
C45	−0.0281 (3)	0.5374 (5)	0.3944 (3)	0.0347 (13)	
H46	−0.0195	0.4852	0.4312	0.042*	
C46	−0.0878 (3)	0.5947 (5)	0.3726 (3)	0.0392 (15)	
H47	−0.1206	0.5820	0.3947	0.047*	
C47	−0.1001 (3)	0.6699 (5)	0.3190 (4)	0.0441 (16)	
H48	−0.1413	0.7087	0.3042	0.053*	
C48	−0.0525 (3)	0.6894 (5)	0.2866 (3)	0.0388 (15)	
H49	−0.0618	0.7409	0.2494	0.047*	
C49	0.0269 (2)	0.7526 (4)	0.1944 (3)	0.0260 (11)	
C50	−0.0080 (3)	0.7093 (5)	0.1318 (3)	0.0320 (13)	
H50	−0.0092	0.6272	0.1236	0.038*	
C51	−0.0407 (3)	0.7846 (5)	0.0820 (3)	0.0353 (13)	
H51	−0.0639	0.7534	0.0397	0.042*	
C52	−0.0407 (3)	0.9047 (5)	0.0917 (3)	0.0338 (13)	
H52	−0.0622	0.9560	0.0564	0.041*	
C53	−0.0087 (3)	0.9477 (5)	0.1540 (3)	0.0347 (14)	
H53	−0.0096	1.0296	0.1622	0.042*	
C54	0.0249 (2)	0.8734 (4)	0.2051 (3)	0.0283 (12)	
H54	0.0466	0.9051	0.2477	0.034*	
P5	0.54660 (9)	0.74420 (15)	0.06486 (10)	0.0454 (4)	
F1	0.5545 (6)	0.6026 (9)	0.0426 (6)	0.050 (3)*	0.40
F2	0.6147 (7)	0.7668 (12)	0.0489 (8)	0.080 (4)*	0.40
F3	0.5868 (8)	0.6925 (13)	0.1387 (9)	0.099 (5)*	0.40
F4	0.5469 (10)	0.8632 (12)	0.0914 (8)	0.093 (4)*	0.40
F5	0.4805 (7)	0.7031 (11)	0.0818 (8)	0.061 (4)*	0.40
F6	0.5053 (7)	0.7599 (12)	−0.0126 (7)	0.063 (4)*	0.40
F7	0.4865 (10)	0.8345 (18)	0.0424 (11)	0.091 (6)*	0.30
F8	0.5342 (11)	0.7245 (15)	0.1337 (10)	0.075 (6)*	0.30
F9	0.5920 (7)	0.8583 (10)	0.1011 (7)	0.040 (3)*	0.30
F10	0.6126 (8)	0.6707 (13)	0.0894 (9)	0.062 (4)*	0.30
F11	0.5743 (9)	0.7863 (13)	0.0019 (9)	0.063 (4)*	0.30
F12	0.5083 (10)	0.6387 (15)	0.0333 (10)	0.084 (5)*	0.30
F13	0.4740 (8)	0.6838 (14)	0.0584 (10)	0.053 (5)*	0.30
F14	0.5076 (7)	0.8755 (11)	0.0654 (8)	0.040 (3)*	0.30
F15	0.5624 (9)	0.7389 (13)	0.1454 (8)	0.043 (4)*	0.30
F16	0.6071 (8)	0.8210 (16)	0.0700 (10)	0.073 (5)*	0.30
F17	0.5809 (9)	0.6314 (14)	0.0656 (9)	0.056 (4)*	0.30
F18	0.5308 (11)	0.7675 (15)	−0.0113 (9)	0.057 (5)*	0.30

### Atomic displacement parameters (Å^2^)

**Table:**

	*U*^11^	*U*^22^	*U*^33^	*U*^12^	*U*^13^	*U*^23^
Ru1	0.01844 (17)	0.02342 (17)	0.0212 (2)	−0.00033 (19)	0.00615 (14)	0.00016 (19)
S1	0.0202 (7)	0.0355 (8)	0.0317 (10)	0.0002 (6)	0.0031 (7)	−0.0012 (6)
N1	0.030 (2)	0.036 (2)	0.025 (3)	0.003 (2)	0.009 (2)	0.005 (2)
C1	0.032 (3)	0.051 (4)	0.036 (4)	−0.005 (3)	−0.005 (3)	0.001 (3)
C2	0.041 (3)	0.042 (3)	0.029 (4)	0.003 (3)	0.002 (3)	0.007 (3)
P1	0.0218 (6)	0.0257 (6)	0.0225 (8)	−0.0015 (5)	0.0071 (6)	0.0001 (6)
C3	0.029 (3)	0.026 (2)	0.028 (3)	0.001 (2)	0.010 (2)	0.005 (2)
C4	0.027 (3)	0.031 (3)	0.029 (3)	−0.004 (2)	0.013 (3)	0.001 (2)
P2	0.0249 (7)	0.0241 (6)	0.0241 (8)	−0.0012 (5)	0.0078 (6)	−0.0014 (6)
C5	0.016 (2)	0.031 (3)	0.031 (3)	0.001 (2)	0.008 (2)	0.002 (2)
C6	0.025 (3)	0.028 (3)	0.026 (3)	−0.002 (2)	0.007 (2)	−0.001 (2)
C7	0.037 (3)	0.040 (3)	0.027 (3)	−0.008 (2)	0.007 (3)	−0.007 (3)
C8	0.046 (4)	0.049 (4)	0.023 (4)	−0.007 (3)	0.007 (3)	−0.004 (3)
C9	0.023 (3)	0.048 (3)	0.023 (3)	−0.003 (2)	0.002 (2)	0.007 (3)
C10	0.025 (3)	0.035 (3)	0.031 (3)	−0.006 (2)	0.012 (3)	0.002 (2)
C11	0.023 (2)	0.022 (2)	0.029 (3)	−0.001 (2)	0.009 (2)	0.002 (2)
C12	0.028 (3)	0.051 (3)	0.027 (4)	−0.004 (3)	0.013 (3)	−0.002 (3)
C13	0.031 (3)	0.063 (4)	0.032 (4)	0.004 (3)	0.015 (3)	0.012 (3)
C14	0.024 (3)	0.043 (3)	0.051 (4)	0.004 (2)	0.017 (3)	0.010 (3)
C15	0.024 (3)	0.045 (3)	0.052 (4)	−0.007 (3)	0.009 (3)	−0.008 (3)
C16	0.026 (3)	0.043 (3)	0.033 (4)	0.001 (2)	0.006 (3)	−0.004 (3)
C17	0.036 (3)	0.026 (3)	0.031 (3)	0.004 (2)	0.014 (3)	−0.006 (2)
C18	0.038 (3)	0.037 (3)	0.045 (4)	0.002 (3)	0.004 (3)	0.000 (3)
C19	0.032 (3)	0.040 (3)	0.067 (5)	0.010 (3)	0.008 (3)	0.008 (3)
C20	0.029 (3)	0.050 (4)	0.056 (5)	0.012 (3)	0.007 (3)	−0.009 (3)
C21	0.034 (3)	0.047 (4)	0.048 (4)	−0.004 (3)	0.018 (3)	−0.003 (3)
C22	0.025 (3)	0.034 (3)	0.034 (4)	0.005 (2)	0.007 (3)	0.001 (2)
C23	0.039 (3)	0.020 (2)	0.029 (3)	0.002 (2)	0.004 (3)	0.000 (2)
C24	0.037 (3)	0.026 (3)	0.039 (4)	−0.001 (2)	0.005 (3)	0.000 (3)
C25	0.037 (3)	0.042 (3)	0.036 (4)	0.004 (3)	−0.005 (3)	−0.006 (3)
C26	0.053 (4)	0.034 (3)	0.032 (4)	0.004 (3)	−0.006 (3)	−0.008 (3)
C27	0.067 (5)	0.043 (3)	0.023 (4)	0.013 (3)	0.007 (3)	−0.004 (3)
C28	0.042 (3)	0.030 (3)	0.027 (3)	0.005 (2)	0.007 (3)	−0.003 (2)
P3	0.0215 (6)	0.0231 (6)	0.0253 (8)	0.0010 (5)	0.0069 (6)	0.0006 (6)
C29	0.030 (3)	0.022 (2)	0.032 (3)	−0.002 (2)	0.009 (3)	0.000 (2)
C30	0.022 (3)	0.029 (3)	0.032 (3)	−0.001 (2)	0.011 (2)	0.001 (2)
P4	0.0194 (7)	0.0261 (6)	0.0254 (9)	−0.0009 (6)	0.0064 (6)	0.0002 (6)
C31	0.026 (3)	0.030 (3)	0.027 (3)	0.012 (2)	0.008 (2)	0.002 (2)
C32	0.039 (3)	0.053 (4)	0.033 (4)	−0.001 (3)	0.009 (3)	−0.009 (3)
C33	0.053 (4)	0.059 (4)	0.034 (4)	0.012 (3)	0.018 (3)	−0.013 (3)
C34	0.049 (4)	0.067 (4)	0.039 (4)	0.024 (3)	0.023 (3)	0.005 (4)
C35	0.039 (4)	0.045 (4)	0.059 (5)	0.013 (3)	0.027 (3)	0.005 (3)
C36	0.032 (3)	0.029 (3)	0.041 (4)	0.004 (2)	0.013 (3)	0.003 (3)
C37	0.036 (3)	0.028 (3)	0.029 (4)	0.004 (2)	0.013 (3)	0.003 (2)
C38	0.046 (3)	0.029 (3)	0.041 (4)	0.002 (2)	0.018 (3)	0.011 (3)
C39	0.061 (4)	0.034 (3)	0.049 (5)	0.011 (3)	0.023 (4)	0.017 (3)
C40	0.071 (5)	0.036 (3)	0.031 (4)	0.018 (3)	0.012 (4)	0.007 (3)
C41	0.054 (4)	0.037 (3)	0.043 (4)	0.017 (3)	0.010 (3)	0.008 (3)
C42	0.038 (3)	0.038 (3)	0.041 (4)	0.011 (3)	0.010 (3)	0.008 (3)
C43	0.021 (3)	0.028 (3)	0.031 (3)	−0.001 (2)	0.009 (2)	0.001 (2)
C44	0.028 (3)	0.036 (3)	0.035 (4)	−0.003 (2)	0.014 (3)	0.000 (3)
C45	0.039 (3)	0.036 (3)	0.033 (4)	−0.008 (2)	0.018 (3)	−0.002 (3)
C46	0.035 (3)	0.043 (3)	0.051 (4)	−0.009 (3)	0.031 (3)	−0.004 (3)
C47	0.029 (3)	0.052 (4)	0.056 (5)	0.009 (3)	0.020 (3)	0.006 (3)
C48	0.024 (3)	0.046 (3)	0.053 (4)	0.008 (2)	0.021 (3)	0.011 (3)
C49	0.015 (2)	0.032 (3)	0.030 (3)	−0.004 (2)	0.006 (2)	0.001 (2)
C50	0.025 (3)	0.038 (3)	0.032 (3)	−0.004 (2)	0.006 (3)	−0.002 (3)
C51	0.024 (3)	0.047 (3)	0.031 (4)	0.001 (2)	0.001 (3)	0.007 (3)
C52	0.024 (3)	0.045 (3)	0.029 (3)	−0.001 (2)	0.000 (2)	0.011 (3)
C53	0.029 (3)	0.029 (3)	0.048 (4)	0.002 (2)	0.014 (3)	0.011 (3)
C54	0.023 (3)	0.031 (3)	0.029 (3)	−0.003 (2)	0.004 (2)	0.003 (2)
P5	0.0505 (10)	0.0507 (10)	0.0380 (11)	−0.0106 (8)	0.0173 (9)	−0.0057 (8)

### Geometric parameters (Å, º)

**Table:**

Ru1—N1	2.209 (5)	C28—H30	0.9500
Ru1—P1	2.3586 (14)	P3—C31	1.835 (5)
Ru1—P4	2.3672 (15)	P3—C37	1.838 (6)
Ru1—P3	2.3698 (13)	P3—C29	1.850 (5)
Ru1—P2	2.4249 (13)	C29—C30	1.517 (7)
Ru1—S1	2.4317 (15)	C29—H31	0.9900
S1—C1	1.831 (7)	C29—H32	0.9900
N1—C2	1.484 (8)	C30—P4	1.849 (5)
N1—H1	0.90 (2)	C30—H33	0.9900
N1—H2	0.91 (2)	C30—H34	0.9900
C1—C2	1.478 (8)	P4—C49	1.837 (6)
C1—H3	0.9900	P4—C43	1.856 (5)
C1—H4	0.9900	C31—C32	1.386 (8)
C2—H5	0.9900	C31—C36	1.406 (7)
C2—H6	0.9900	C32—C33	1.404 (9)
P1—C5	1.842 (6)	C32—H35	0.9500
P1—C11	1.859 (5)	C33—C34	1.358 (9)
P1—C3	1.860 (5)	C33—H36	0.9500
C3—C4	1.517 (8)	C34—C35	1.372 (9)
C3—H7	0.9900	C34—H37	0.9500
C3—H8	0.9900	C35—C36	1.379 (8)
C4—P2	1.833 (5)	C35—H38	0.9500
C4—H9	0.9900	C36—H39	0.9500
C4—H10	0.9900	C37—C42	1.390 (8)
P2—C23	1.834 (6)	C37—C38	1.407 (8)
P2—C17	1.835 (5)	C38—C39	1.397 (8)
C5—C10	1.396 (7)	C38—H40	0.9500
C5—C6	1.403 (7)	C39—C40	1.366 (9)
C6—C7	1.386 (8)	C39—H41	0.9500
C6—H11	0.9500	C40—C41	1.375 (10)
C7—C8	1.378 (8)	C40—H42	0.9500
C7—H12	0.9500	C41—C42	1.388 (8)
C8—C9	1.377 (8)	C41—H43	0.9500
C8—H13	0.9500	C42—H44	0.9500
C9—C10	1.389 (8)	C43—C48	1.395 (7)
C9—H14	0.9500	C43—C44	1.396 (8)
C10—H15	0.9500	C44—C45	1.389 (7)
C11—C16	1.388 (8)	C44—H45	0.9500
C11—C12	1.389 (8)	C45—C46	1.380 (8)
C12—C13	1.385 (8)	C45—H46	0.9500
C12—H16	0.9500	C46—C47	1.376 (9)
C13—C14	1.373 (9)	C46—H47	0.9500
C13—H17	0.9500	C47—C48	1.386 (8)
C14—C15	1.375 (9)	C47—H48	0.9500
C14—H18	0.9500	C48—H49	0.9500
C15—C16	1.396 (8)	C49—C54	1.398 (7)
C15—H19	0.9500	C49—C50	1.400 (8)
C16—H20	0.9500	C50—C51	1.375 (8)
C17—C22	1.391 (7)	C50—H50	0.9500
C17—C18	1.403 (7)	C51—C52	1.384 (8)
C18—C19	1.369 (8)	C51—H51	0.9500
C18—H21	0.9500	C52—C53	1.378 (8)
C19—C20	1.375 (9)	C52—H52	0.9500
C19—H22	0.9500	C53—C54	1.390 (8)
C20—C21	1.377 (9)	C53—H53	0.9500
C20—H23	0.9500	C54—H54	0.9500
C21—C22	1.381 (7)	P5—F4	1.466 (14)
C21—H24	0.9500	P5—F17	1.475 (14)
C22—H25	0.9500	P5—F12	1.495 (17)
C23—C24	1.396 (8)	P5—F16	1.531 (16)
C23—C28	1.405 (8)	P5—F8	1.552 (19)
C24—C25	1.375 (8)	P5—F18	1.555 (18)
C24—H26	0.9500	P5—F10	1.585 (15)
C25—C26	1.374 (9)	P5—F2	1.592 (13)
C25—H27	0.9500	P5—F7	1.601 (19)
C26—C27	1.390 (9)	P5—F5	1.610 (13)
C26—H28	0.9500	P5—F6	1.617 (14)
C27—C28	1.384 (8)	P5—F15	1.623 (15)
C27—H29	0.9500		
			
N1—Ru1—P1	171.03 (13)	C25—C26—H28	120.5
N1—Ru1—P4	95.14 (13)	C27—C26—H28	120.5
P1—Ru1—P4	91.27 (5)	C28—C27—C26	120.6 (6)
N1—Ru1—P3	87.17 (13)	C28—C27—H29	119.7
P1—Ru1—P3	99.77 (5)	C26—C27—H29	119.7
P4—Ru1—P3	83.88 (5)	C27—C28—C23	121.0 (6)
N1—Ru1—P2	88.63 (13)	C27—C28—H30	119.5
P1—Ru1—P2	83.76 (5)	C23—C28—H30	119.5
P4—Ru1—P2	103.62 (5)	C31—P3—C37	97.8 (2)
P3—Ru1—P2	171.71 (5)	C31—P3—C29	105.9 (3)
N1—Ru1—S1	80.92 (13)	C37—P3—C29	102.8 (3)
P1—Ru1—S1	93.86 (5)	C31—P3—Ru1	123.39 (17)
P4—Ru1—S1	169.03 (5)	C37—P3—Ru1	116.92 (18)
P3—Ru1—S1	85.70 (5)	C29—P3—Ru1	107.81 (16)
P2—Ru1—S1	86.59 (5)	C30—C29—P3	110.2 (3)
C1—S1—Ru1	101.18 (19)	C30—C29—H31	109.6
C2—N1—Ru1	116.7 (4)	P3—C29—H31	109.6
C2—N1—H1	104 (4)	C30—C29—H32	109.6
Ru1—N1—H1	118 (4)	P3—C29—H32	109.6
C2—N1—H2	99 (4)	H31—C29—H32	108.1
Ru1—N1—H2	118 (4)	C29—C30—P4	111.0 (4)
H1—N1—H2	98 (5)	C29—C30—H33	109.4
C2—C1—S1	114.3 (4)	P4—C30—H33	109.4
C2—C1—H3	108.7	C29—C30—H34	109.4
S1—C1—H3	108.7	P4—C30—H34	109.4
C2—C1—H4	108.7	H33—C30—H34	108.0
S1—C1—H4	108.7	C49—P4—C30	101.7 (3)
H3—C1—H4	107.6	C49—P4—C43	100.6 (2)
C1—C2—N1	112.9 (5)	C30—P4—C43	98.9 (2)
C1—C2—H5	109.0	C49—P4—Ru1	123.42 (16)
N1—C2—H5	109.0	C30—P4—Ru1	107.70 (17)
C1—C2—H6	109.0	C43—P4—Ru1	120.52 (19)
N1—C2—H6	109.0	C32—C31—C36	119.3 (5)
H5—C2—H6	107.8	C32—C31—P3	121.7 (4)
C5—P1—C11	99.3 (2)	C36—C31—P3	118.7 (4)
C5—P1—C3	101.4 (2)	C31—C32—C33	119.6 (6)
C11—P1—C3	99.4 (2)	C31—C32—H35	120.2
C5—P1—Ru1	123.58 (17)	C33—C32—H35	120.2
C11—P1—Ru1	121.42 (18)	C34—C33—C32	120.1 (6)
C3—P1—Ru1	107.59 (18)	C34—C33—H36	120.0
C4—C3—P1	110.8 (4)	C32—C33—H36	120.0
C4—C3—H7	109.5	C33—C34—C35	120.8 (6)
P1—C3—H7	109.5	C33—C34—H37	119.6
C4—C3—H8	109.5	C35—C34—H37	119.6
P1—C3—H8	109.5	C34—C35—C36	120.6 (6)
H7—C3—H8	108.1	C34—C35—H38	119.7
C3—C4—P2	110.4 (3)	C36—C35—H38	119.7
C3—C4—H9	109.6	C35—C36—C31	119.5 (5)
P2—C4—H9	109.6	C35—C36—H39	120.3
C3—C4—H10	109.6	C31—C36—H39	120.3
P2—C4—H10	109.6	C42—C37—C38	117.6 (5)
H9—C4—H10	108.1	C42—C37—P3	120.1 (4)
C4—P2—C23	101.5 (3)	C38—C37—P3	122.2 (4)
C4—P2—C17	104.3 (2)	C39—C38—C37	119.6 (6)
C23—P2—C17	100.2 (3)	C39—C38—H40	120.2
C4—P2—Ru1	106.29 (18)	C37—C38—H40	120.2
C23—P2—Ru1	120.91 (17)	C40—C39—C38	121.2 (6)
C17—P2—Ru1	121.00 (17)	C40—C39—H41	119.4
C10—C5—C6	117.8 (5)	C38—C39—H41	119.4
C10—C5—P1	122.4 (4)	C39—C40—C41	120.1 (6)
C6—C5—P1	119.7 (4)	C39—C40—H42	119.9
C7—C6—C5	121.3 (5)	C41—C40—H42	119.9
C7—C6—H11	119.4	C40—C41—C42	119.4 (6)
C5—C6—H11	119.4	C40—C41—H43	120.3
C8—C7—C6	119.7 (5)	C42—C41—H43	120.3
C8—C7—H12	120.1	C41—C42—C37	122.0 (6)
C6—C7—H12	120.1	C41—C42—H44	119.0
C9—C8—C7	120.1 (6)	C37—C42—H44	119.0
C9—C8—H13	119.9	C48—C43—C44	116.9 (5)
C7—C8—H13	119.9	C48—C43—P4	121.4 (4)
C8—C9—C10	120.5 (5)	C44—C43—P4	121.6 (4)
C8—C9—H14	119.7	C45—C44—C43	122.5 (5)
C10—C9—H14	119.7	C45—C44—H45	118.8
C9—C10—C5	120.6 (5)	C43—C44—H45	118.8
C9—C10—H15	119.7	C46—C45—C44	118.8 (5)
C5—C10—H15	119.7	C46—C45—H46	120.6
C16—C11—C12	117.8 (5)	C44—C45—H46	120.6
C16—C11—P1	119.2 (4)	C47—C46—C45	120.4 (5)
C12—C11—P1	123.0 (4)	C47—C46—H47	119.8
C13—C12—C11	121.3 (6)	C45—C46—H47	119.8
C13—C12—H16	119.3	C46—C47—C48	120.3 (5)
C11—C12—H16	119.3	C46—C47—H48	119.9
C14—C13—C12	120.5 (6)	C48—C47—H48	119.9
C14—C13—H17	119.8	C47—C48—C43	121.2 (6)
C12—C13—H17	119.8	C47—C48—H49	119.4
C13—C14—C15	119.1 (5)	C43—C48—H49	119.4
C13—C14—H18	120.4	C54—C49—C50	117.7 (5)
C15—C14—H18	120.4	C54—C49—P4	119.2 (4)
C14—C15—C16	120.7 (6)	C50—C49—P4	123.0 (4)
C14—C15—H19	119.7	C51—C50—C49	120.5 (5)
C16—C15—H19	119.7	C51—C50—H50	119.8
C11—C16—C15	120.6 (6)	C49—C50—H50	119.8
C11—C16—H20	119.7	C50—C51—C52	121.9 (6)
C15—C16—H20	119.7	C50—C51—H51	119.1
C22—C17—C18	117.0 (5)	C52—C51—H51	119.1
C22—C17—P2	119.1 (4)	C53—C52—C51	118.0 (5)
C18—C17—P2	123.9 (4)	C53—C52—H52	121.0
C19—C18—C17	121.3 (6)	C51—C52—H52	121.0
C19—C18—H21	119.3	C52—C53—C54	121.1 (5)
C17—C18—H21	119.3	C52—C53—H53	119.4
C18—C19—C20	120.4 (6)	C54—C53—H53	119.4
C18—C19—H22	119.8	C53—C54—C49	120.7 (5)
C20—C19—H22	119.8	C53—C54—H54	119.7
C19—C20—C21	119.9 (5)	C49—C54—H54	119.7
C19—C20—H23	120.0	F17—P5—F16	95.6 (11)
C21—C20—H23	120.0	F12—P5—F8	94.6 (10)
C20—C21—C22	119.6 (5)	F17—P5—F18	97.0 (9)
C20—C21—H24	120.2	F16—P5—F18	84.6 (11)
C22—C21—H24	120.2	F12—P5—F10	92.0 (9)
C21—C22—C17	121.7 (5)	F8—P5—F10	89.5 (9)
C21—C22—H25	119.2	F4—P5—F2	91.4 (8)
C17—C22—H25	119.2	F12—P5—F7	95.8 (11)
C24—C23—C28	116.7 (5)	F8—P5—F7	92.0 (10)
C24—C23—P2	122.0 (5)	F10—P5—F7	171.9 (10)
C28—C23—P2	121.1 (4)	F4—P5—F5	95.4 (8)
C25—C24—C23	122.2 (6)	F2—P5—F5	172.3 (7)
C25—C24—H26	118.9	F4—P5—F6	102.7 (9)
C23—C24—H26	118.9	F2—P5—F6	92.0 (8)
C26—C25—C24	120.5 (6)	F5—P5—F6	89.8 (8)
C26—C25—H27	119.7	F17—P5—F15	90.1 (9)
C24—C25—H27	119.7	F16—P5—F15	91.6 (10)
C25—C26—C27	118.9 (6)	F18—P5—F15	172.2 (8)
			
N1—Ru1—S1—C1	1.0 (2)	C25—C26—C27—C28	2.8 (9)
P1—Ru1—S1—C1	173.7 (2)	C26—C27—C28—C23	−2.4 (9)
P4—Ru1—S1—C1	−68.6 (4)	C24—C23—C28—C27	1.0 (8)
P3—Ru1—S1—C1	−86.8 (2)	P2—C23—C28—C27	176.4 (4)
P2—Ru1—S1—C1	90.2 (2)	N1—Ru1—P3—C31	−152.5 (2)
P4—Ru1—N1—C2	−169.9 (4)	P1—Ru1—P3—C31	21.8 (2)
P3—Ru1—N1—C2	106.5 (4)	P4—Ru1—P3—C31	112.0 (2)
P2—Ru1—N1—C2	−66.4 (4)	S1—Ru1—P3—C31	−71.4 (2)
S1—Ru1—N1—C2	20.4 (4)	N1—Ru1—P3—C37	−31.4 (2)
Ru1—S1—C1—C2	−22.2 (5)	P1—Ru1—P3—C37	142.8 (2)
S1—C1—C2—N1	41.0 (6)	P4—Ru1—P3—C37	−126.9 (2)
Ru1—N1—C2—C1	−40.8 (6)	S1—Ru1—P3—C37	49.7 (2)
P4—Ru1—P1—C5	−23.9 (2)	N1—Ru1—P3—C29	83.6 (2)
P3—Ru1—P1—C5	60.1 (2)	P1—Ru1—P3—C29	−102.1 (2)
P2—Ru1—P1—C5	−127.5 (2)	P4—Ru1—P3—C29	−11.8 (2)
S1—Ru1—P1—C5	146.40 (19)	S1—Ru1—P3—C29	164.7 (2)
P4—Ru1—P1—C11	−153.23 (19)	C31—P3—C29—C30	−95.6 (4)
P3—Ru1—P1—C11	−69.21 (19)	C37—P3—C29—C30	162.4 (4)
P2—Ru1—P1—C11	103.22 (19)	Ru1—P3—C29—C30	38.3 (4)
S1—Ru1—P1—C11	17.08 (19)	P3—C29—C30—P4	−49.2 (5)
P4—Ru1—P1—C3	93.51 (18)	C29—C30—P4—C49	168.7 (4)
P3—Ru1—P1—C3	177.52 (18)	C29—C30—P4—C43	−88.4 (4)
P2—Ru1—P1—C3	−10.05 (18)	C29—C30—P4—Ru1	37.7 (4)
S1—Ru1—P1—C3	−96.19 (18)	N1—Ru1—P4—C49	144.9 (3)
C5—P1—C3—C4	169.2 (4)	P1—Ru1—P4—C49	−28.8 (2)
C11—P1—C3—C4	−89.1 (4)	P3—Ru1—P4—C49	−128.5 (2)
Ru1—P1—C3—C4	38.2 (4)	P2—Ru1—P4—C49	55.1 (2)
P1—C3—C4—P2	−51.7 (4)	S1—Ru1—P4—C49	−146.7 (4)
C3—C4—P2—C23	168.0 (4)	N1—Ru1—P4—C30	−97.4 (2)
C3—C4—P2—C17	−88.2 (4)	P1—Ru1—P4—C30	88.91 (19)
C3—C4—P2—Ru1	40.8 (4)	P3—Ru1—P4—C30	−10.79 (19)
N1—Ru1—P2—C4	161.9 (2)	P2—Ru1—P4—C30	172.79 (19)
P1—Ru1—P2—C4	−13.36 (19)	S1—Ru1—P4—C30	−29.0 (4)
P4—Ru1—P2—C4	−103.16 (19)	N1—Ru1—P4—C43	14.7 (2)
S1—Ru1—P2—C4	80.90 (19)	P1—Ru1—P4—C43	−159.01 (19)
N1—Ru1—P2—C23	47.2 (3)	P3—Ru1—P4—C43	101.30 (19)
P1—Ru1—P2—C23	−128.0 (2)	P2—Ru1—P4—C43	−75.12 (19)
P4—Ru1—P2—C23	142.2 (2)	S1—Ru1—P4—C43	83.1 (4)
S1—Ru1—P2—C23	−33.8 (2)	C37—P3—C31—C32	86.9 (5)
N1—Ru1—P2—C17	−79.7 (3)	C29—P3—C31—C32	−18.9 (5)
P1—Ru1—P2—C17	105.1 (2)	Ru1—P3—C31—C32	−143.6 (4)
P4—Ru1—P2—C17	15.3 (2)	C37—P3—C31—C36	−87.8 (5)
S1—Ru1—P2—C17	−160.6 (2)	C29—P3—C31—C36	166.5 (4)
C11—P1—C5—C10	−98.7 (4)	Ru1—P3—C31—C36	41.8 (5)
C3—P1—C5—C10	3.0 (5)	C36—C31—C32—C33	−1.0 (9)
Ru1—P1—C5—C10	123.3 (4)	P3—C31—C32—C33	−175.6 (5)
C11—P1—C5—C6	79.1 (4)	C31—C32—C33—C34	2.6 (10)
C3—P1—C5—C6	−179.2 (4)	C32—C33—C34—C35	−2.9 (11)
Ru1—P1—C5—C6	−58.9 (4)	C33—C34—C35—C36	1.5 (10)
C10—C5—C6—C7	−1.0 (7)	C34—C35—C36—C31	0.1 (10)
P1—C5—C6—C7	−178.9 (4)	C32—C31—C36—C35	−0.3 (9)
C5—C6—C7—C8	1.0 (8)	P3—C31—C36—C35	174.4 (5)
C6—C7—C8—C9	−0.1 (9)	C31—P3—C37—C42	43.7 (5)
C7—C8—C9—C10	−0.7 (9)	C29—P3—C37—C42	152.1 (5)
C8—C9—C10—C5	0.7 (8)	Ru1—P3—C37—C42	−90.1 (5)
C6—C5—C10—C9	0.1 (7)	C31—P3—C37—C38	−139.2 (5)
P1—C5—C10—C9	178.0 (4)	C29—P3—C37—C38	−30.8 (6)
C5—P1—C11—C16	164.8 (4)	Ru1—P3—C37—C38	87.0 (5)
C3—P1—C11—C16	61.4 (5)	C42—C37—C38—C39	−1.4 (9)
Ru1—P1—C11—C16	−56.0 (5)	P3—C37—C38—C39	−178.6 (5)
C5—P1—C11—C12	−13.1 (5)	C37—C38—C39—C40	1.0 (10)
C3—P1—C11—C12	−116.4 (5)	C38—C39—C40—C41	−0.8 (10)
Ru1—P1—C11—C12	126.1 (4)	C39—C40—C41—C42	1.1 (10)
C16—C11—C12—C13	0.0 (9)	C40—C41—C42—C37	−1.6 (10)
P1—C11—C12—C13	177.9 (5)	C38—C37—C42—C41	1.7 (9)
C11—C12—C13—C14	−1.3 (10)	P3—C37—C42—C41	179.0 (5)
C12—C13—C14—C15	0.3 (10)	C49—P4—C43—C48	−2.9 (5)
C13—C14—C15—C16	1.8 (9)	C30—P4—C43—C48	−106.6 (5)
C12—C11—C16—C15	2.1 (8)	Ru1—P4—C43—C48	136.7 (4)
P1—C11—C16—C15	−175.8 (4)	C49—P4—C43—C44	174.3 (4)
C14—C15—C16—C11	−3.1 (9)	C30—P4—C43—C44	70.5 (5)
C4—P2—C17—C22	162.9 (5)	Ru1—P4—C43—C44	−46.1 (5)
C23—P2—C17—C22	−92.3 (5)	C48—C43—C44—C45	−1.5 (8)
Ru1—P2—C17—C22	43.5 (6)	P4—C43—C44—C45	−178.7 (4)
C4—P2—C17—C18	−16.6 (6)	C43—C44—C45—C46	0.7 (9)
C23—P2—C17—C18	88.2 (5)	C44—C45—C46—C47	0.2 (9)
Ru1—P2—C17—C18	−136.1 (5)	C45—C46—C47—C48	−0.3 (10)
C22—C17—C18—C19	−0.1 (9)	C46—C47—C48—C43	−0.6 (10)
P2—C17—C18—C19	179.5 (5)	C44—C43—C48—C47	1.4 (9)
C17—C18—C19—C20	−0.2 (11)	P4—C43—C48—C47	178.7 (5)
C18—C19—C20—C21	1.1 (11)	C30—P4—C49—C54	−174.5 (4)
C19—C20—C21—C22	−1.8 (10)	C43—P4—C49—C54	84.0 (4)
C20—C21—C22—C17	1.5 (10)	Ru1—P4—C49—C54	−54.0 (5)
C18—C17—C22—C21	−0.6 (9)	C30—P4—C49—C50	7.9 (5)
P2—C17—C22—C21	179.8 (5)	C43—P4—C49—C50	−93.6 (5)
C4—P2—C23—C24	−47.9 (5)	Ru1—P4—C49—C50	128.4 (4)
C17—P2—C23—C24	−155.0 (4)	C54—C49—C50—C51	2.8 (7)
Ru1—P2—C23—C24	69.2 (5)	P4—C49—C50—C51	−179.6 (4)
C4—P2—C23—C28	136.9 (4)	C49—C50—C51—C52	−0.4 (8)
C17—P2—C23—C28	29.8 (5)	C50—C51—C52—C53	−2.2 (8)
Ru1—P2—C23—C28	−106.0 (4)	C51—C52—C53—C54	2.4 (8)
C28—C23—C24—C25	−0.2 (8)	C52—C53—C54—C49	0.0 (8)
P2—C23—C24—C25	−175.5 (4)	C50—C49—C54—C53	−2.6 (7)
C23—C24—C25—C26	0.7 (9)	P4—C49—C54—C53	179.7 (4)
C24—C25—C26—C27	−1.9 (9)		

### Hydrogen-bond geometry (Å, º)

**Table:**

*D*—H···*A*	*D*—H	H···*A*	*D*···*A*	*D*—H···*A*
N1—H1···F11^i^	0.90 (2)	2.34 (4)	3.198 (17)	159 (6)

Symmetry code: (i) *x*−1/2, −*y*+3/2, *z*+1/2.
